# Using eye tracking to assess learning of a multifunction prosthetic hand: an exploratory study from a rehabilitation perspective

**DOI:** 10.1186/s12984-024-01445-3

**Published:** 2024-08-31

**Authors:** Wendy Hill, Helen Lindner

**Affiliations:** 1 Institute of Biomedical Engineering, University of New Brunswick, Fredericton, Canada; 2 School of Health Sciences, Faculty of Medicine and Health, Örebro University, 701 82 Örebro, Sweden

**Keywords:** Multifunction prosthetic hand, Training, Eye tracking, Fixation, Saccade, Rehabilitation

## Abstract

**Background:**

Eye tracking technology not only reveals the acquisition of visual information at fixation but also has the potential to unveil underlying cognitive processes involved in learning to use a multifunction prosthetic hand. It also reveals gaze behaviours observed during standardized tasks and self-chosen tasks. The aim of the study was to explore the use of eye tracking to track learning progress of multifunction hands at two different time points in prosthetic rehabilitation.

**Methods:**

Three amputees received control training of a multifunction hand with new control strategy. Detailed description of control training was collected first. They wore Tobii Pro2 eye-tracking glasses and performed a set of standardized tasks (required to switch to different grips for each task) after one day of training and at one-year-follow-up (missing data for Subject 3 at the follow up due to socket problem). They also performed a self-chosen task (free to use any grip for any object) and were instructed to perform the task in a way how they would normally do at home. The gaze-overlaid videos were analysed using the Tobii Pro Lab and the following metrics were extracted: fixation duration, saccade amplitude, eye-hand latency, fixation count and time to first fixation.

**Results:**

During control training, the subjects learned 3 to 4 grips. Some grips were easier, and others were more difficult because they forgot or were confused with the switching strategies. At the one-year-follow-up, a decrease in performance time, fixation duration, eye-hand latency, and fixation count was observed in Subject 1 and 2, indicating an improvement in the ability to control the multifunction hand and a reduction of cognitive load. An increase in saccade amplitude was observed in both subjects, suggesting a decrease in difficulty to control the prosthetic hand. During the standardized tasks, the first fixation of all three subjects were on the multifunction hand in all objects. During the self-chosen tasks, the first fixations were mostly on the objects first.

**Conclusion:**

The qualitative data from control training and the quantitative eye tracking data from clinical standardized tasks provided a rich exploration of cognitive processing in learning to control a multifunction hand. Many prosthesis users prefer multifunction hands and with this study we have demonstrated that a targeted prosthetic training protocol with reliable assessment methods will help to lay the foundation for measuring functional benefits of multifunction hands.

**Supplementary Information:**

The online version contains supplementary material available at 10.1186/s12984-024-01445-3.

## Introduction

Increased dexterity in multifunction prosthetic hands offers hope to upper limb (UL) amputees that have been previously using a single degree of freedom (DOF) prosthetic hand. At the same time, using multifunction hands begins with a journey of learning that can be cognitively demanding. This learning process can be explained using the model of skill acquisition proposed by Fitts and Posner, which includes three stages: cognitive, associative, and autonomous stages [1]. At the cognitive stage, the prosthesis users learn the technical parts of the device and how to control it. At the associative stage, attention may be focused on specific details, such as grip switching. At the autonomous stage, the user keeps practising until his/her performance enters an automatized routine. Prosthesis users gradually learn to use different prosthetic grips effectively to hold  everyday objects of different materials, weights and sizes.

Since the commercial introduction of the first multifunction prosthetic hand in 2007 and its first publication [2], significant advancements have been made in both functionality and aesthetics. These include features like intelligent finger sensing, improved grip options, a more robust movable thumb, and a multi-position wrist [3, 4]. While prosthesis users generally prefer the enhanced functions and appearance of multifunction hands over other terminal device options such as single DOF hands [5, 6], conflicting findings are reported regarding their functional benefits [5, 7–9]. Additionally, questions arise around the cost-effectiveness of multifunction hands compared to single DOF prosthetic hands [10]. It is crucial to underscore the importance of targeted training for users of multifunction hands to achieve optimal functional outcomes [5, 7, 10].

During training, therapists closely monitor the user's progress with the prosthesis and tailor the pace and direction of training to ensure success [11]. One way to help monitor the progress in learning multifunction hands is to use clinical outcome measures. Between 2017 and 2023, a number of observational or self-reported clinical tools have been used to assess functional outcomes of multifunction hands and control strategies (Table 1).

**Table 1:** Clinical outcome measures that have been used to assess multifunction hands between 2017 and 2023

Clinical outcome measures	Studies between 2017 and 2023
Prosthetic hand assessment/questionnaires	
Activities Measure for Upper Limb Amputee*	Resnik [8], Resnik [12], Simon [9]
Assessment of Capacity for Myoelectric Control*	Widehammar [5], Yu [13], Simon [9]
Brief Activity Measure for Upper Limb Amputees	Resnik [14]
Capacity Assessment of Prosthetic Performance for the Upper Limb	Kearns [15]
Patient Specific Functional Scale*	Resnik [8]
Refined clothespin relocation test	Kerver [7], Hussaini [16]
Southampton Hand Assessment Procedure*	Widehammar[5] Kerver [7], Resnik [17], Simon [9]
Upper Extremity Functional Scale*	Kerver [7], Yu [13]
University of New Brunswick Test of Prosthetic Function: Spontaneity*	Resnik [8]
Timed activity performance in persons with upper limb amputation*	Resnik [18]
Trinity amputation and prosthesis experience scales for upper extremity*	Kerver [7], Yu [13]
Human hand assessment	
Action research arm test	Salminger [19]
Box and blocks test	Kerver [7], Yu [13], Simon [9]
Jebsen-Taylor Hand Function Test	Yu [13], Simon [9]
Tray test	Kerver [7]
Pain and satisfaction assessment	
Quebec User Evaluation of Satisfaction with assistive technology	Kerver [7]
Disabilities of the Arm, Shoulder, and Hand score	Resnik [20]
Visual Analogue Scale/Short-Form McGill Pain Questionnaire	Yu [13]

*Psychometrically tested with UL amputees

Some functional assessments were originally developed for single DOF prosthetic hands whereas others were developed for human hand function. It is thus questionable whether the study results were valid for multifunctional hands [5, 8, 9]. For example, the dexterity measures in one study did not observe any differences between multifunction hands and single DOF hands. Functional assessments for single DOF hands may not adequately capture the improved dexterity of multifunction hands. Objective measures that assess functional outcomes of multifunction hands are thus crucial for prosthetic rehabilitation. These measures, particularly those that capture cognitive or mental processing during the use of multifunction hands, provide valuable information for therapists to adjust training pace effectively.

In recent years, eye tracking has been suggested to reveal cognitive processes in neuroscience research [21]. The interpretation of gaze behavior is commonly based on the eye-mind hypothesis, in which it is assumed that the eyes fixate on the entity with which the mind is engaged [22, 23]. Eye gaze metrics such as longer fixation duration on special areas during task performance have been demonstrated to be signs for engagement or mental processing [24]. Saccade amplitude decreases as task difficulty increases and the need to gather more fine-grained information increases [25]. Eye tracking metrics hence have the potential to measure cognitive processes in learning to use multifunction hands. For example, switching between different grips in multifunction hands is not intuitive and can be cognitively demanding [26, 27].

A recent review suggested that eye tracking technology can be an effective tool to quantitatively assess visuomotor behaviour among single DOF prosthesis users and able-bodied controls [28]. Based on the 17 included studies, the review concluded that visual attention was directed more towards the prosthetic hand and less towards the target during object manipulation tasks. However, most of these studies used standardized tasks under experimental conditions, which limits the transferability of this finding to self-chosen everyday tasks. Therapists frequently evaluate prosthesis users using self-chosen tasks because the ultimate goal of prosthetic rehabilitation is to empower amputees to seamlessly incorporate the multifunction hand into their self-chosen everyday tasks.

In terms of gaze behavior and single DOF prostheses, several studies showed a large hand-focused gaze during prosthesis use [29–31] and it took longer to disengage gaze from manipulations to plan upcoming movements [30]. When training was given, the hand-focused gaze on the single DOF prosthesis decreased in one study but remained the same in another study [29, 31]. It is unknown whether hand-focused gaze would decrease in users who are learning to use multifunction hands due to the additional complexity of multiple grip patterns and grip switching methods.

With this study, we explore the use of eye tracking to track learning progress of multifunction hands at two different time points over one year. The following research questions are explored in this study: (i) can gaze measurements be used to track learning progress in multifunction hands? (ii) how do gaze and use behaviors differ in standardized versus self-chosen tasks?

## Methods

### Design

An exploratory design was used to explore gaze behavior in two different conditions: while performing standardized tasks and self-chosen tasks using multiple grip patterns in a multifunction prosthetic hand.

### Subjects

A convenience sample of three UL amputees (2 right-handed, all male, mean age 47.6) were recruited from the Atlantic Clinic for Upper Limb Prosthetics clinic at the University of New Brunswick, Canada. Inclusion criteria were that subjects were using a myoelectric hand, either conventional (one DOF) or multifunction hand with any control system, that they were at least 19 years old, and that they were able to return to the clinic for follow-up testing. The Research Ethics Board of University of New Brunswick approved the study (REB 2019–009) and all subjects signed informed consent forms before participating in the study.

All subjects recruited for the study lost their dominant hand (2 right and 1 left) after workplace accidents (Appendix 1, Table 4). They had previously experienced direct control of a myoelectric prosthetic device before being enrolled in this study and all were being fitted with a new multifunction hand or a new control system for operating the hand. In this study, Subject 1 and 2 used gesture control for switching grips but Subject 2 used Coapt Pattern Recognition for hand open and close. Similarly, Subject 3 used Coapt Pattern Recognition to control multiple grip patterns of the hand as well as a powered wrist.

### Training

All subjects went through basic skills training of learning to open and close the hand, and switch between three to four basic grips and use them to pick up and manipulate objects of varying shapes and sizes. The specific grips used depended on the type of hand being used and the control system being used. For instance, Subject 1 and 2 had the iLimb Quantum hand using gesture control where Subject 3 had a TASKA hand and used pattern recognition to control wrist rotation, hand open, and pinch, key, and power grip. Gesture control allows for grips to be assigned to four different directions of movement. The user must hold an open signal and then move in one of four directions; forward, back, left, or right to access the desired grip. If more options were available for control of the hand, more grips were trained during this initial skills training period.

**Fig. 1:**
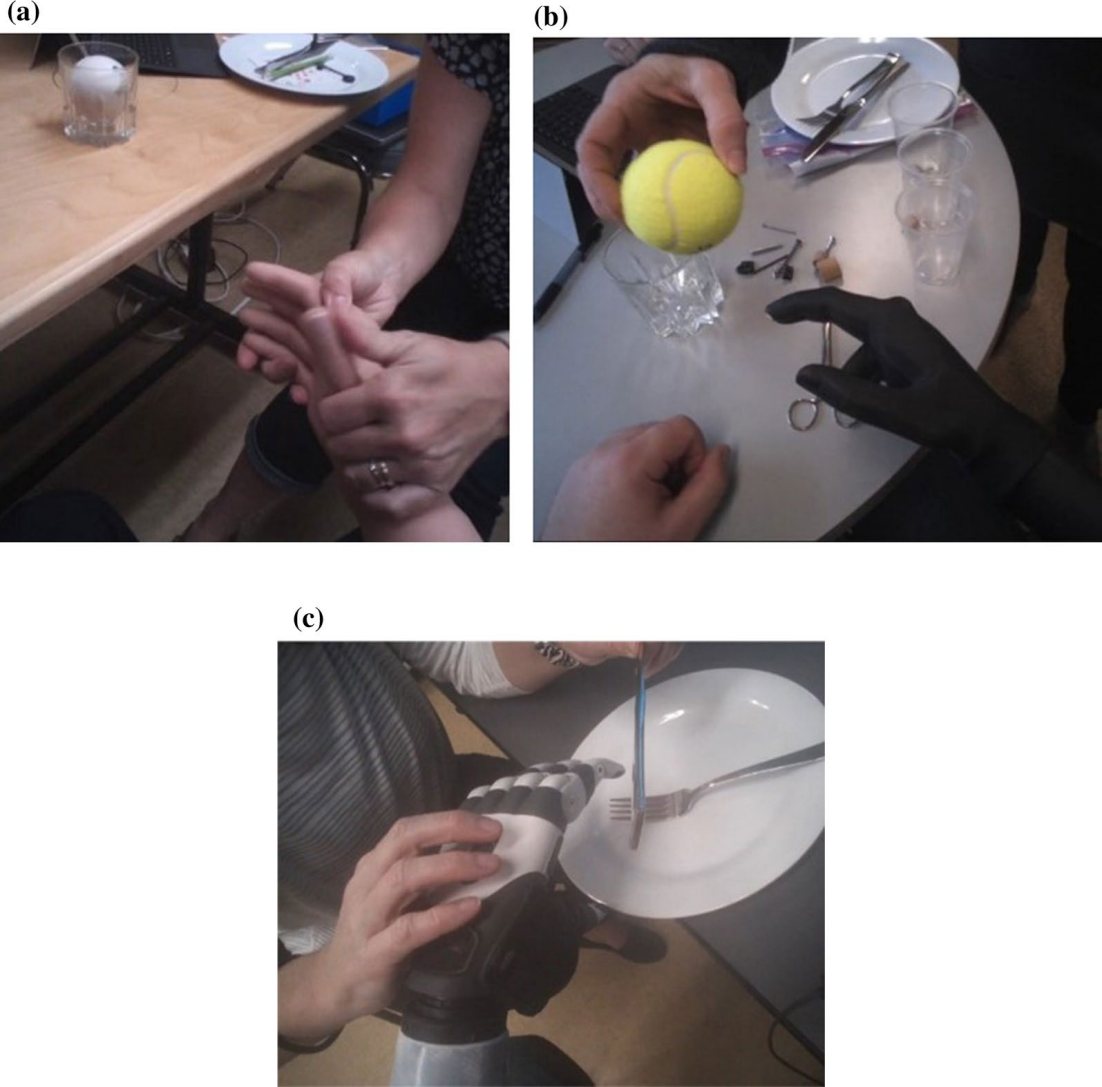
**a** Subject 1: Learning to switch to power grip for a glass. **b** Subject 2: Learning to switch from tripod grip to power grip for a tennis ball. **c** Subject 3: Learning to switch from power grip to lateral grip to hold a knife

Skills training included tasks such as: grasping a tennis ball with a spherical grip and putting it down, moving one-inch blocks using a tripod grip, opening a reusable plastic bag using a tripod or pinch grip, holding a drinking glass with a cylindrical grip while pouring water, picking up small objects with a tripod or pinch grip, holding a dinner plate with a lateral grip, and cutting food with fork and knife using a lateral grip (Fig. 1a–c).

### Standardized tasks and self-chosen tasks

**Fig. 2:**
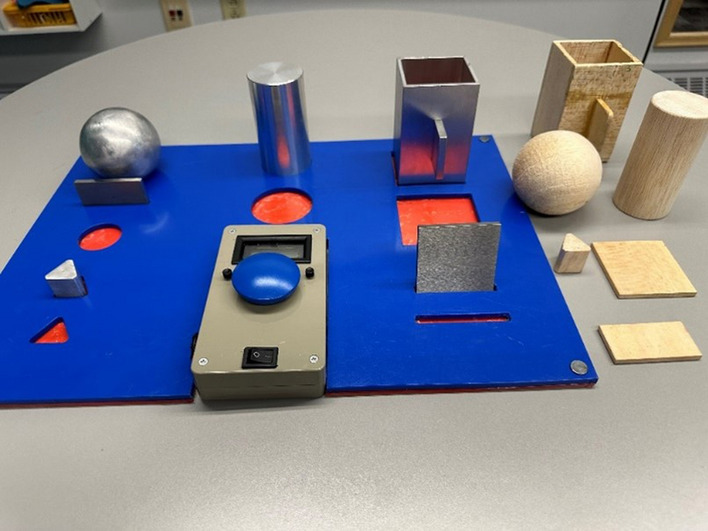
The standardized tasks—the two sets of abstract objects of different weights (wood and metal) in the Southampton Hand Assessment Procedure

The standardized tasks are one part of the *Southampton Hand Assessment Procedure* (SHAP). It includes moving 12 abstract objects using six different hand grasps: spherical, tripod, power, lateral, tip and extension [17, 32] (Fig. 2). Performance of each task is timed by the subject. The SHAP is performed in a seated position with the multifunction hand in a neutral hand and wrist position at the start.

**Fig. 3:**
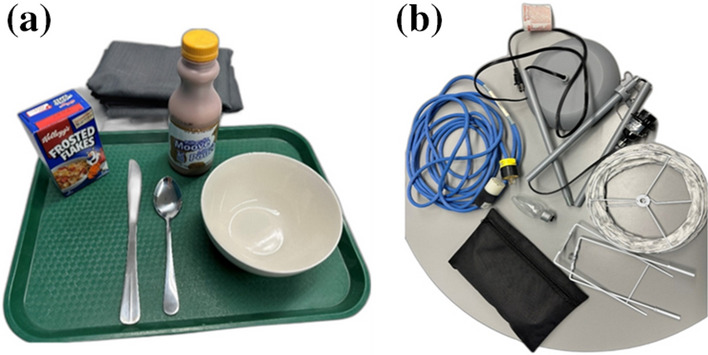
**a** and **b**. The self-chosen tasks—breakfast task and assemble a lamp task

The self-chosen tasks are tasks that are used in the *Assessment of Capacity for Myoelectric Control* (ACMC) [11]. The ACMC assesses movement quality (instead of time completion) using self-chosen tasks. For this study, we only used the ACMC self-chosen tasks for observation purposes. The subjects are free to choose any grip to perform the self-chosen task. The objects are daily common ADL objects, such as utensils, milk bottle, light bulb, lamp stand etc. (Fig. 3a and b). The users are encouraged to perform the self-chosen task in their usual way and at their own pace.

### Eye-tracking measurement

Binocular movements were recorded using Tobii Pro Glasses 2 at a sampling rate of 50 Hz (every 20 ms). The head-mounted eye tracker is a video-based eye-tracking system that records gaze movements of the wearer continuously during use. The system records the gaze positions on the x- and y axis at a sampling frequency of at 50 Hz, while the video is recorded with a 1920 × 1080 px resolution at 25 frames per second [33].

### Procedure for gaze data collection

The subjects were asked to wear the Tobii Pro glasses with the appropriate nose pad. Calibration was performed using the built-in one-point target calibration procedure. After calibration, they performed the SHAP using the multifunction hand at baseline (after 1 day training) and at a follow-up visit within one year. All gaze measurements were taken in the same activity room without windows. A table was placed at the same location under a fluorescent ceiling lamp. During the SHAP, the patient was in a seated position with the table set to the appropriate height to allow the elbow to rest at 90 degrees on the table surface. During the self-chosen task, the subject performed the activity while moving around a room, reaching into cupboards and spaces of different heights. Lighting was not consistent during the self-chosen task. The self-chosen tasks were performed after the performance of SHAP.

### Data analysis

The eye tracker recordings were imported in the Tobii Pro Lab [34]. The recordings were first inspected to remove unexpected eye movements due to sudden head movements. Area of interest (AOI) and time of interest (TOI) were identified. The multifunction hands and the objects were labelled as different AOIs. The TOI in the SHAP standardized tasks is the time to complete a SHAP object movement. The TOI in the ACMC self-chosen tasks is the time from reaching for one object to just before the prosthetic hand was reaching for the next object.

Four eye tracking metrics, that are commonly used to assess cognitive processes, were chosen to answer research question 1 [35]. Fixation duration measures the temporal aspect of visual behaviour, indicating how long attention is maintained on a specific target [35]. In contrast, spatial measures in eye tracking typically refer to characteristics such as saccade amplitudes (the distance between successive fixations) or fixation count distribution across different areas of interest. In Table 2, we presented the four chosen metrics definitions and their relevance to learning to use a multifunction hand. For research question 2, we used the time to first fixation on either the multifunction hand or the objects to indicate gaze behaviour between standardized and self-chosen tasks.

**Table 2:** Eye tracking metrics and their relevance in learning multifunction hands

Metrics	Definition	Learning multifunction hand
*For Research Question 1: can gaze measurements be used to track learning progress in multifunction hands?*		
Fixation duration (seconds)	Indicates how long the eyes fixate on an object during a TOI [35]	Fixation duration increases when processing becomes more effortful [24, 36],such as the time taken to mentally choose or activate the correct muscles/gestures for a desired grip
Fixation count (frequency)	The number of times the eye fixates on a particular object [35]	A relative high number of fixations indicates a relative high degree of attentional activity takes place, e.g. a high degree of visual feedback is needed during grasping, holding or releasing objects [11]
Eye-hand latency (seconds)	The duration from the start of a fixation on an object until the hand performs the action associated with the object [35]	How soon after the eye looks at an object does the multifunction hand grasp the object? When switching grip is easier, the latency is shorter
Saccade amplitude (degree)	The angular distance the eye travels during the movement [35]	The saccade amplitude decreases with increasing difficulty or cognitive load [37], e.g. stare at the multifunction hand when trying to get the desired grip
*For Research Question 2: how do gaze and use behaviors differ in standardized versus self-chosen tasks?*		
Time to first fixation (seconds)	The time taken from stimulus onset up to the first fixation into a particular AOI [35]	Indicates the time to first fixation (in seconds) for hand versus objects in standardized and self-chosen tasks

Using the Pro Lab, heat maps were generated to show fixation durations on the multifunction hand. Deep red represents long fixation duration; green represents short fixation duration.

## Results

### Control training

Subjects 1 and 2 learned four different grips whereas Subject 3 learned three different grips (Table 3). Key grip was an easy grip for Subject 1 and 3 whereas it was a difficult grip for Subject 2. Power grip was easy for Subject 2, but it was a difficult grip for Subject 1 and Subject 3. Various reasons contributed to their difficulty, such as Subject 1 had a problem remembering direction in gesture control, and Subject 3 was confused with the pinch grip and power grip in Pattern Recognition. Subject 2 had previously used an iLimb Ultra hand and developed a habit of manually positioning the thumb to change the grip from oppositional to lateral, so gesture control was a significant change in control strategy.

### Prosthetic wear time

Subject 1 and 3 wore the prosthetic hand for around 2 and 3–4 h per day respectively (Table 4). Subject 2 wore the prosthetic hand for 9–10 h per day.

### Performance time for standardized tasks

Table 5 (the first row) shows the performance time of the standardized tasks at two different time points. After 1 day of training, Subject 1 took 422.58 s to complete the twelve tasks. The power grip for the heavy metal ball took 75.08 s (the longest time). At the one-year follow-up, Subject 1 had an average of 35% decrease in performance time.

After 1 day of training, Subject 2 took 212.33 s to complete the twelve tasks and the key grip for the wooden box took 110.08 s (the longest time). At the one-year follow-up, Subject 2 had an average of 60% decrease in performance time.

### Eye tracking metrics during standardized tasks

**Fig. 4:**
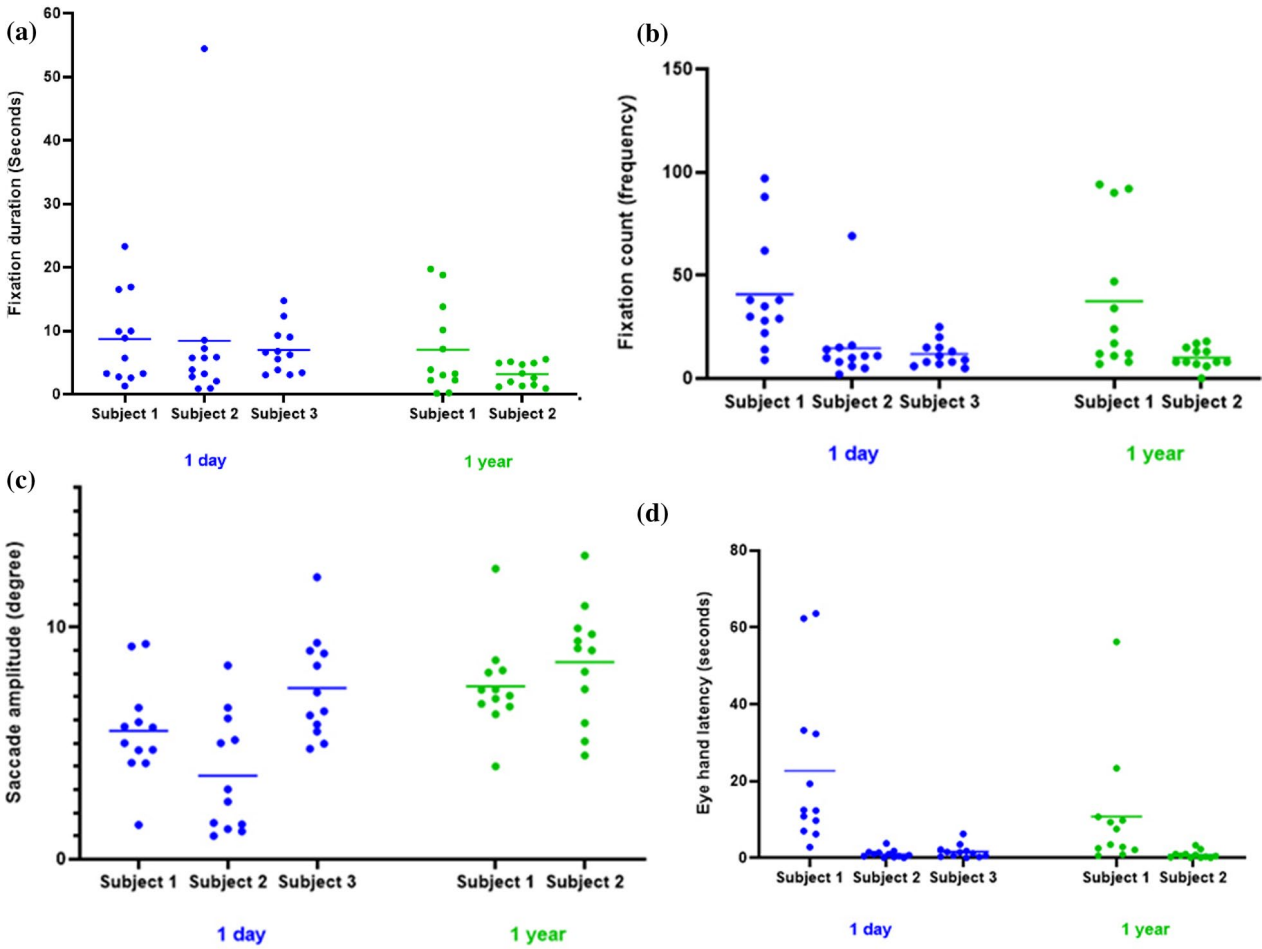
**a**–**d** Fixation duration, fixation count, saccade amplitude and eye hand latency of the 3 subjects. Each dot is one object in the standardised task (total 12 objects)

Table 5 (Appendix 1) also shows total and average gaze measurements during standardized tasks at two different time points. Figure 4 (a,b,c,d) shows the gaze measurements for individual SHAP objects  at two different points. After one day of training, Subject 1 had the longest fixation duration, highest fixation count and longest eye-hand latency among all three subjects. In terms of saccade amplitude, Subject 2 had the lowest saccade amplitude among all three subjects, indicating that cognitive load was highest in Subject 2 during the SHAP standardized tasks.

At the one-year follow-up, both Subject 1 and 2 showed a decrease in fixation duration, fixation count and eye-hand latency. An increase in saccade amplitude was observed in both Subject 1 and 2, indicating that it was less mentally demanding for them to control the multifunction hand. Due to socket problem, Subject 3 was not able to operate the multifunction hand at the follow-up appointment and hence no data was collected at that visit.

**Fig. 5:**
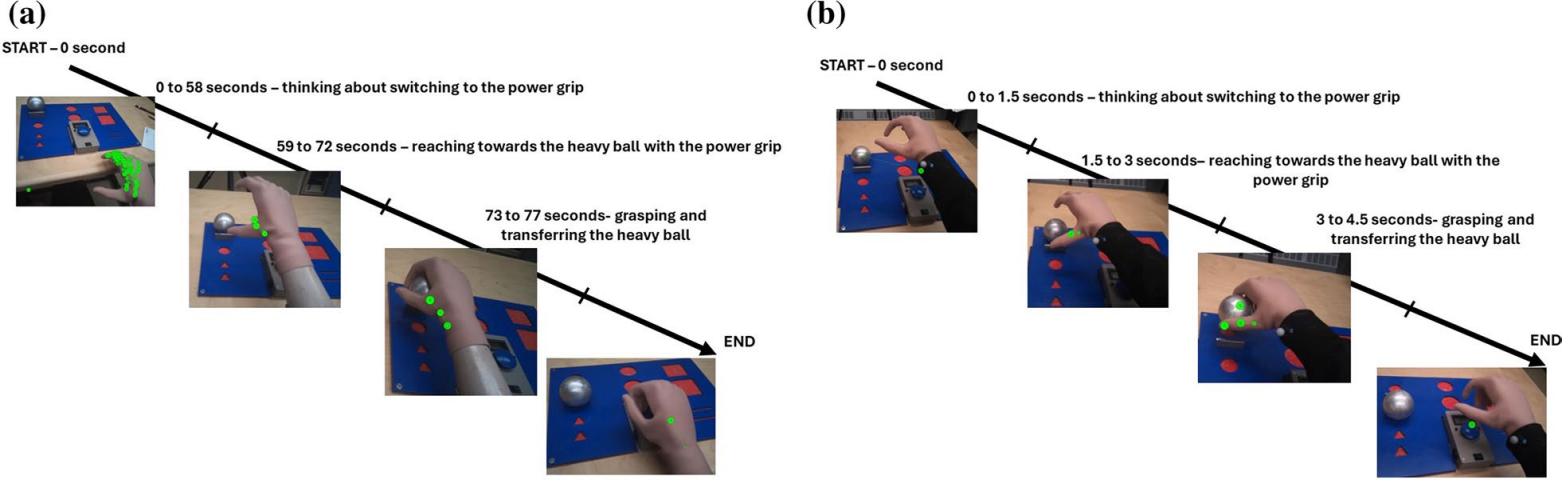
**a** and **b** Subject 1- gaze behaviour of power grip after 1 day and at 1 year

For Subject 1, the longest fixation duration, highest fixation count, lowest saccade amplitude and longest eye-hand latency were during thinking about grip switching for power grip (for a spherical object), pinch grip (for a tip object) and tripod grip (for a triangle object). For example, the gaze fixation count (numbers of green dots) and fixation duration (size of the green dots) for the power grip are shown after one day training (Fig. 5a) and at one year (Fig. 5b) (also see the videos under supplementary material). After one day training, Subject 1 took 58 s with high numbers of fixation counts and fixation duration to think about switching to the power grip. His fixations were clustered on  the multifunction hand, which led to a relative low saccade amplitude.

**Fig. 6:**
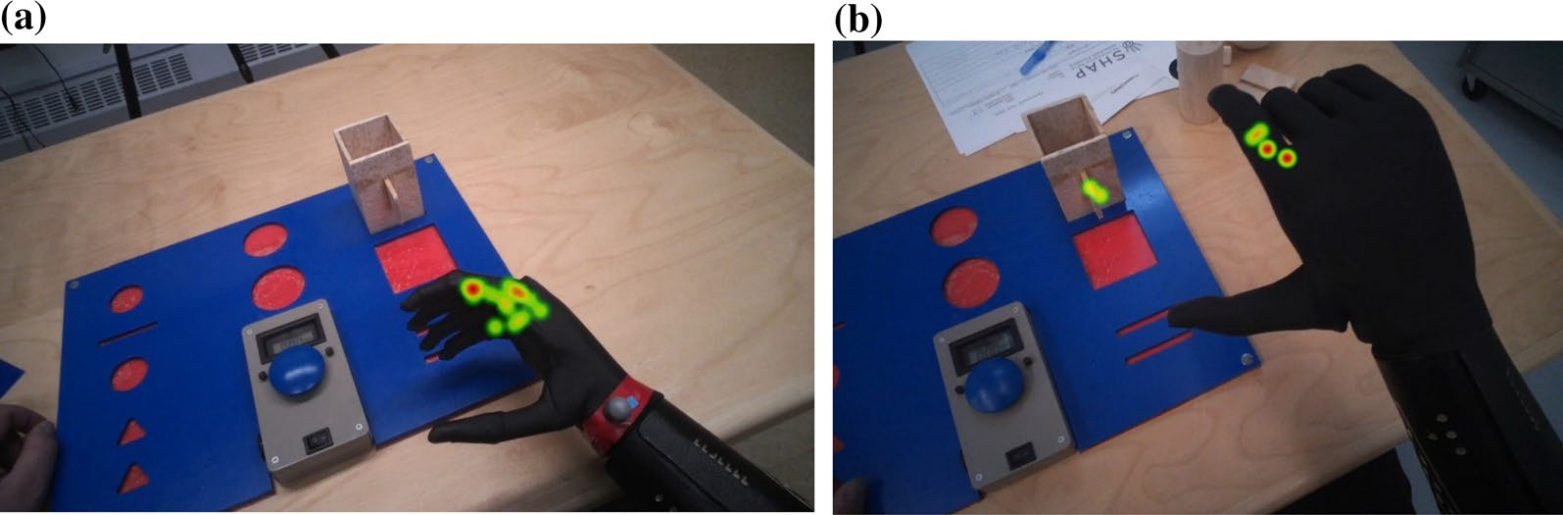
**a** and **b** Heat maps of Subject 2 thinking about switching to lateral grip for the wooden box after 1 day training (time taken: 15 s) and after 1 year (time taken: 4 s)

For Subject 2, the longest fixation duration, highest fixation count, lowest saccade amplitude and longest eye-hand latency were during grip switching into the lateral grip (Fig. 6a). Both Subject 1 and 2 have improved in accessing the difficult grips at 1 year follow up (Figs. 5b and 6b).

**Fig. 7:**
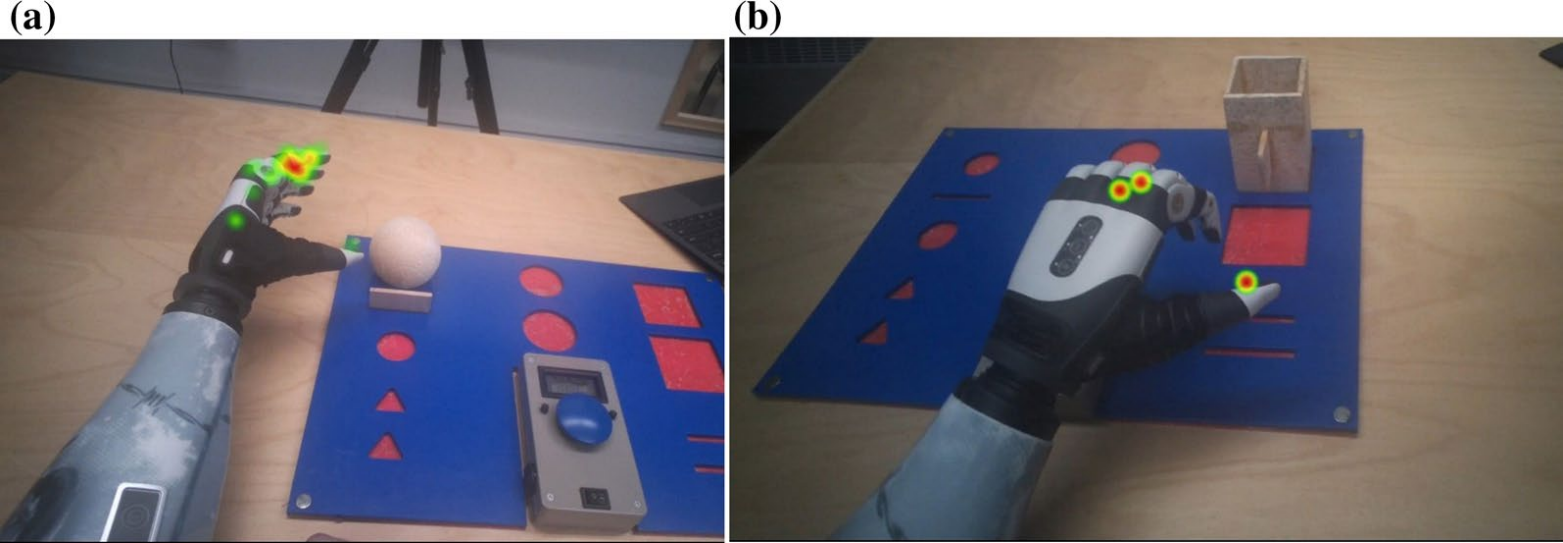
**a** and **b** Heat maps of Subject 3 thinking about switching to power grip for the wooden ball (Time taken: 36 s) and switching to lateral grip for the wooden box (time taken: 4 s) after 1 day training

For Subject 3, the longest fixation duration, highest fixation count, lowest saccade amplitude and longest eye-hand latency was during grip switching into the power grip (Fig. 7a) and during grip switching into a lateral grip (Fig. 7b).

### Differences in gaze and use behaviors in standardized versus self-chosen tasks

#### Frequency of changing grips

In the standardized tasks, the subjects were required to switch from a neutral hand position to a desired grip for grasping two sets of 6 objects of different shapes. In the self-chosen tasks, the subjects were allowed to use any grip they liked. Subject 1 changed grips at two different times in the self-chosen task (once for grasping the cereal box with the power grip and once for grasping the tablecloth with the pinch grip). Subject 2 changed grips at 4 different times (pinch grip for the cable on two occasions, tripod grip for the light bulb on two different occasions). Subject 3 changed grips 21 times for grasping all different objects at different occasions.

#### Time to first fixation for hand versus objects

Table 6 (Appendix 1) shows the time to first fixation on the multifunction hand and  on the objects. During the standardized tasks, the first fixation of all three subjects were on the multifunction hand in all objects. During the self-chosen tasks, the first fixations were mostly on the objects first.

## Discussion

The present study explored the use of both qualitative data from control training and quantitative eye tracking data from clinical standardized tasks to understand and measure cognitive processing and gaze behaviour in learning to control a multifunction hand. Two different multifunction hands and two kinds of control strategies were being used. Two subjects showed improvements, with a decrease in performance time, fixation duration, fixation count and eye hand latency.

To our best knowledge, this is the first multifunction hand study that combines the data from clinical control training and eye tracking data from clinical standardized tasks. Compared with the number of studies on prosthetic components, literature on learning to control a multifunction hand in a clinical setting is sparse. This detailed report of control training and clinical findings are from a therapist perspective. Traditionally, therapist researchers perform research in prosthetic training whereas engineers perform research in prosthetic technologies [38]. The findings of control training provide valuable information on the importance of control strategies for multifunction hands. For example, as shown in Fig. 5a, the high fixation counts, long fixation durations and low saccade amplitude of Subject 1 occurred when he was thinking about switching to the power grip using gesture control. His fixation counts and durations were similar after he had switched to the power grip. This may suggest that thinking about the control of the multifunction hand is more cognitively demanding than the action of moving the hand. Similarly, Subject 2 was thinking about switching between two different grips when he showed the lowest saccade amplitude. An intuitive control strategy may contribute to the ease of controlling the prosthetic hand with multiple  degree of freedom.

Previous studies using eye tracking metrics were investigated in able-bodied individuals or amputees using single DOF prostheses [28–31, 39]. In this study, we aim to delve deeper into the use of gaze measurements among users of prosthetic hands that have multiple DOF prosthetic hands. The findings in fixation duration during the standardized tasks are consistent with previous studies on single DOF prostheses [29–31]. After one day of training, the prosthetic users were still in the cognitive process of understanding the new hand and learning to control it. At the follow-up testing, both Subject 1 and 2 showed a 30% and 60% decrease in fixation duration respectively. This suggests that they were moving towards the autonomous stage, as proposed by the learning theory by Fitts and Posner [1].

In contrast to previous studies, our study is the first to explore cognitive processing using fixation duration and saccade amplitude on UL amputees. Previous studies had used fixation duration to indicate visual attention, skill level and spatiotemporal disruption in able-bodied individuals [29–31, 39]. In the neuroscience field, fixation duration has been suggested to surpass pupil size as a measure of memory load [24]. Pupil size was not a reliable measure for self-chosen real-life tasks because real life tasks are seldom performed in one position and the pupil size will be affected by different light conditions.

Saccade amplitude is the distance travelled by the eye between two fixation points and it increases if the difficulty decreases [25]. All subjects learned 3 to 4 grips during control training, and they had been practising in their home environment. Similarly, both subjects also showed a 24% and 138% increase respectively in saccade amplitude. After one day of training, both subjects stared at the hand (i.e. one position) during grip switching. At the one-year follow-up, however, they looked around at different objects and the hand during the SHAP standardized tasks. Although more research using other outcome measures with a large sample is needed, our findings on shorter fixation durations and larger saccade amplitudes at 1 year are likely attributed to reduced mental processing or memory load, indicating lesser difficulty in controlling the multifunction hands after one year. From a learning perspective, as proposed by Fitts and Posner [1], during the autonomous stage, the user keeps practising using their prosthesis in familiar self-chosen daily tasks until his/her performance enters an automatized routine. An earlier study showed that full time prosthetic users (8 h or more) acquired the highest ability to control single DOF prostheses [40]. Subject 1 used his multifunction hand for 2 h per day whereas Subject 2 used his hand for 9–10 h per day. Subject 1's relatively low improvement, compared to Subject 2, may be attributed to his limited 2 h of actual use of the prosthetic hand.

Eye-hand latency is well-investigated in developmental neuroscience because it provides valuable insights into the coordination between visual processing and motor responses. Similar to previous studies [30, 41], a decrease in eye-hand latency was observed in both Subject 1 and 2 during the one-year- follow up, suggesting that a stronger coupling between visual processing and prosthesis use existed at the one-year follow-up than after one day of training. When there is a strong coupling between visual processing and prosthesis use, it means that the subjects were effectively utilizing visual feedback to guide and adjust their prosthetic grips [30].

Fixation count on the prosthetic hand is a spatial measure that shows the distribution of fixations. From clinical experience, we know that new users look at their hands during operation and when they become experienced users, the need for visual feedback will be much less [42]. In our subjects, we observed high fixation counts with long fixation durations after one day of training, which may indicate sustained attention of the multifunction hand. A decrease in fixation count at the one-year follow-up suggests the need for visual feedback has decreased.

The second question we asked was “how do gaze and use behaviors differ in standardized versus self-chosen tasks?”. Previous studies using standardized tasks showed that the gaze of able-bodied individuals and prosthesis users of single DOF were mostly on the prosthesis [28–31, 41]. Our findings showed that users of multifunction hands first fixated on the objects in the self-chosen tasks whereas their fixation first on the hand in the standardized tasks. This gaze difference has an important implication for assessment purposes. Standardized tasks are good for research purposes but if the purpose is to measure real life functional benefits, self-chosen tasks will provide a more valid assessment in terms of visual attention.

In terms of use behaviour, the frequency of grip switching during self-chosen tasks was much lower in Subject 1 compared to Subject 2 and 3. One possible reason could be that Subject 1 used gesture control and previous research suggested that gesture control requires too much time for functional grip switching [27]. Subject 3 used pattern recognition as a control strategy and he switched grips multiple times during the self-chosen task. The pattern recognition control system offers the ability to control multiple movements in a relatively seamless manner [43] and it may contribute to the ease of switching grips in Subject 3, although a larger sample with a pre-post design using other outcome measures is needed to further investigate the benefit of different control strategies.

### Clinical implications

Despite being an exploratory study with 3 subjects, we would like to address several implications here. Clinically, we notice patients tend to stick to a limited number of grips in real-life situations and self-chosen tasks. Gaze measurement data supports this observation, indicating that switching grips is more cognitively demanding. While standardized tasks may show improved grip switching with practice, in self-chosen tasks, patients often refrain from frequent grip changes. We speculate that prosthesis users often develop habits based on the control method or hand they learned first (for example, in Subject 2, he learned to manually position the thumb to switch grips), or that certain control strategies require more effort or are time-consuming (like gesture control), leading them to stick to one or two basic hand grips. Their focus appears to be more on completing a given task rather than controlling the hand in specific ways. This reflects a more accurate picture of how they use their hands at home.

Using eye tracking metrics, we have shown grip switching is cognitively demanding, yet this skill can improve with time. Grip switching is a new skill for amputees with experience of single DOF prostheses. Functional tests serve as valuable tools, offering a swift assessment of our clients’ abilities. However, functional tests have limitations, such as the SHAP test demonstrates floor effect [17], and eye tracking metrics may not be bounded by floor effect although more research with a diversity of prosthesis users are needed to investigate this.

### Methodological considerations

This exploratory study had 3 subjects and we have demonstrated the use of eye tracking to measure changes in control of multifunction hands. While eye tracking glasses offer valuable insights into visual behavior and cognitive processes, they also have several limitations. Although the subjects wore the glasses in both standardized tasks and in self-chosen tasks, we chose to present fixation and saccade metrics in standardized tasks only. This was because it was difficult to obtain accurate fixation and saccade metrics when the subjects were walking around in the activity room.

Calibration errors or technical issues can affect the reliability of eye tracking data. We did not have any calibration errors; however, the Tobii glasses sometimes stopped in the middle of the recording for unknown reasons. The live view during recording was helpful to notice recording errors.

## Conclusion

With this study, by analyzing gaze behaviors during operation of two types of multifunction hands, we gained insights into mental processing, memory load and attentional processes. By integrating eye tracking into prosthetic rehabilitation programs, clinicians can optimize device control, enhance user experience, and promote successful outcomes of multifunction hands.

### Supplementary Information

Supplementary Material 1.

Supplementary Material 2.

## Data Availability

No datasets were generated or analysed during the current study.

## Appendix 1

See Tables 3, 4, 5, 6

**Table 3:** Hand model, control strategy and training

Subject	Hand model	Control strategy	Training		
			Grips taught	Grips easy for the subject	grips Difficult for the subject
Subject 1	I Limb Quantum	Hold open/gesture control	Spherical/power, key grip, pinch (fingers closed), tripod	Key	Power (had to hold open and not move after the finger twitched), pinch (couldn’t remember direction in gesture control to get to it), tripod (couldn’t remember direction in gesture control)
Subject 2	I Limb Quantum	Pattern recognition for open/close Hold open—gesture control to switch grips	Power, key, tripod, pointer	Power Tripod Pinch	Key grip -time consuming to hold open then had to manually line up thumb with finger to get to desired position More comfortable moving thumb to proper position rather than using gesture control but could get to all positions without problem
Subject 3	TASKA Hand Gen 2	Pattern Recognition to control wrist and hand (direct control of pinch, key, and power grip)	Pinch, key, and Power	Key	Pinch was often confused with Power in pattern recognition

**Table 4:** Subject demographics

	Subject 1	Subject 2	Subject 3
Age	68	33	40
Prosthesis wear time (hrs/day)	2	9–10	3–4
Dominant side/ amputation side	Right/Right	Right/Right	Left/Left
Level of amputation	Transradial	Transhumeral	Transradial
Time since amputation	12 years	10 years	2 years
Previous prosthetic hand(s)	MC Pro-Control, Bebionic	iLimb Ultra (3 years of no use)	OB Greifer
Control of previous hand	Two-site	Two-site (weak muscles)	Two-site
Prosthetic hand assessed	iLimb Quantum	iLimb Quantum	TASKA Hand gen2
Control used	Two-site, gesture control	Coapt pattern recognition for hand open/close, gesture control to switch grips	Coapt Pattern Recognition for control of multiple hand grips

**Table 5:** Performance time and eye tracking metrics during the 12 abstract objects from the Southampton Hand Assessment Procedure

	Subject 1			Subject 2			Subject 3	
	1 day	1 year	Difference	1 day	1 year	Difference	1 day	1 year
Performance time of all 12 tasks (seconds)	422.58 (m = 35.22, *SD* = 23.35)	274.74 (m = 22.90, *SD* = 18.58)	35% less	212.33 (m = 17.68, *SD* = 29.18)	85.34 (m = 7.11, *SD* = 1.19)	60% less	130.64 (m = 10.89, *SD* = 4.36)	–
Total fixation duration on the prosthetic hand (seconds)	104.25 (*m* = *8.69,* *SD* = *7.00*)	84,43 (*m 7.04,* *SD* = *6.99*)	19% less	101.18 (*m* = *8.43,* *SD* = *14.70*)	35.85 (*m* = *3.02,* *SD 1.92*)	65% less	83.76 (*m* = *6.98, SD* = *3.75*)	–
Total fixation count on the prosthetic hand (frequency)	490 (*m* = *40.83,* *SD* = *27.61*)	448 (*m* = *37.33, SD* = *34.92*)	8% less	177 (*m* = *14.75,* *SD 17.58*)	121 (*m* = *10.09, SD* = *5.20*)	32% less	142 (*m* = *11.83, SD* = *6.06*)	No data due to problems with the socket
Eye-hand latency between prosthetic hand and objects (seconds)	271.89 *(m* = *22.66,* *SD* = *21.93)*	129.15 (*m* = *10.76,* *SD* = *15.68*)	52% shorter	12.12 (*m* = *1.01,* *SD* = *1.04*)	9.90 (*m* = *0.82, SD* = *0.99*)	18% shorter	44.80 (*m* = *3.45, SD* = *2.53*)	–
Total saccade amplitude during the tasks (degree)	66.49 *(m* = *5.54,* *SD* = *2.12)*	82.71 (*m* = *8.31,* *SD* = *3.37*)	24% higher*	38.14 (*m* = *3.84,* *SD* = *2.71*)	91.06 (*m* = *8.09,* *SD 2.46*)	138% higher*	88.51 (*m* = *7.38, SD* = *2.20*)	–

*Higher values indicate a wider shift of gaze between objects

**Table 6:** The time to first fixation during standardized tasks and self-chosen tasks

	Subject 1		Subject 2		Subject 3	
	Prosthetic hand	Objects	Prosthetic hand	Objects	Prosthetic hand	Objects
Standardized tasks						
Light abstract objects						
Sphere	0.00	2.86	0.64	1.98	0.00	4.65
Triangle	0.95	57.20	1.22	1.90	0.00	3.60
Power	0.60	23.94	1.22	3.01	0.00	0.94
Lateral	0.00	2.47	0.74	0.74	0.00	2.40
Tip	0.49	11.19	0.7	0.7	0.00	2.55
Extension	0.64	4.10	0.43	1.90	0.00	4.99
Heavy abstract objects						
Sphere	0.00	2.08	0.00	0.19	0.00	2.60
Triangle	0.00	9.28	0.23	0.70	0.00	1.14
Power	0.13	1.00	0.46	0.90	0.00	8.40
Lateral	0.00	9.77	0.00	3.77	0.00	3.06
Tip	0.60	1.15	9.35	10.34	0.26	2.54
Extension	0.50	8.02	0.00	0.98	0.00	2.61
Self-chosen tasks						
Prepare breakfast						
Banana	**2.00**	**1.62**	–	–	–	–
Milk	**2.00**	**0.56**	–	–	–	–
Cornflakes	**4.06**	**2.11**	–	–	–	–
Door handle	**5.76**	**0.00**	–	–	–	–
Spoon	6.95	32.97	–	–	–	–
Plate	6.95	7.97	–	–	–	–
Table cloth	13.36	13.37				
Assemble a table lamp						
Box	–	–	**6.75**	**0.00**	**11.22**	**0.00**
Cable	–	–	**7.93**	**0.00**	**1.52**	**0.00**
Lamp	–	–	**2.68**	**0.86**	**3.06**	**0.00**
Light bulb	–	–	**0.00**	**0.53**	**0.55**	**0.00**

Numbers in bold: the first fixations are on the object first

## References

[1] Fitts P, Posner M. Human performance. Belmont: Brooks/Cole Pub Co; 1967.

[2] Otr OV, et al. The i-LIMB hand and the DMC plus hand compared: a case report. Prosthet Orthot Int. 2010;34(2):216–20. 20470060 10.3109/03093641003767207

[3] Cordella F, et al. Literature review on needs of upper limb prosthesis users. Front Neurosci. 2016;10:209–209. 27242413 10.3389/fnins.2016.00209 PMC4864250

[4] Kyberd PJ. Assessment of functionality of multifunction prosthetic hands. JPO J Prosthet Orthot. 2017;29(3):103–11. 10.1097/JPO.0000000000000139

[5] Widehammar C, et al. Effect of multi-grip myoelectric prosthetic hands on daily activities, pain-related disability and prosthesis use compared with single-grip myoelectric prostheses: a single-case study. J Rehabil Med. 2021. 10.2340/jrm.v53.807. 10.2340/jrm.v53.807 PMC8862656 34766184

[6] Popovic I, et al. Do multi-grip hands increase function and patient satisfaction when compared to traditional myoelectric hands? Can Prosthet Orthot J. 2018. 10.33137/cpoj.v1i2.32049. 10.33137/cpoj.v1i2.32049

[7] Kerver N, et al. The multi-grip and standard myoelectric hand prosthesis compared: does the multi-grip hand live up to its promise? J Neuroeng Rehabil. 2023;20(1):22. 36793049 10.1186/s12984-023-01131-w PMC9930076

[8] Resnik LJ, et al. How do the outcomes of the DEKA Arm compare to conventional prostheses? PLoS ONE. 2018;13(1): e0191326. 29342217 10.1371/journal.pone.0191326 PMC5771605

[9] Simon A, et al. User performance with a transradial multi-articulating hand prosthesis during pattern recognition and direct control home use. IEEE Trans Neural Syst Rehabil Eng. 2022. 10.1109/TNSRE.2022.3221558. 10.1109/TNSRE.2022.3221558 PMC10079638 36355739

[10] Kerver N, et al. Economic evaluation of upper limb prostheses in the Netherlands including the cost-effectiveness of multi-grip versus standard myoelectric hand prostheses. Disabil Rehabil. 2022. 10.1080/09638288.2022.2151653. 36533430 10.1080/09638288.2022.2151653 PMC10721225

[11] Lindner H. The assessment of capacity for myoelectric control: psychometric evidence and comparison with upper limb prosthetic outcome measures. In: Örebro studies in care sciences. Örebro: Örebro Universitet; 2013. p. 85.

[12] Resnik L, et al. Evaluation of EMG pattern recognition for upper limb prosthesis control: a case study in comparison with direct myoelectric control. J Neuroeng Rehabil. 2018;15(1):23. 29544501 10.1186/s12984-018-0361-3 PMC5856206

[13] Yu KE, et al. Clinical evaluation of the revolutionizing prosthetics modular prosthetic limb system for upper extremity amputees. Sci Rep. 2021;11(1):954. 33441604 10.1038/s41598-020-79581-8 PMC7806748

[14] Resnik L, Borgia M, Acluche F. Brief activity performance measure for upper limb amputees: BAM-ULA. Prosthet Orthot Int. 2018;42(1):75–83. 28091278 10.1177/0309364616684196

[15] Kearns NT, et al. Development and psychometric validation of capacity assessment of prosthetic performance for the upper limb (CAPPFUL). Arch Phys Med Rehabil. 2018;99(9):1789–97. 29777713 10.1016/j.apmr.2018.04.021

[16] Hussaini A, Hill W, Kyberd P. Clinical evaluation of the refined clothespin relocation test: a pilot study. Prosthet Orthot Int. 2019;43(5):485–91. 31264508 10.1177/0309364619843779

[17] Resnik L, et al. Psychometric evaluation of the Southampton hand assessment procedure (SHAP) in a sample of upper limb prosthesis users. J Hand Ther. 2023;36(1):110–20. 34400030 10.1016/j.jht.2021.07.003

[18] Resnik L, Borgia M, Acluche F. Timed activity performance in persons with upper limb amputation: a preliminary study. J Hand Ther. 2017;30(4):468–76. 28487130 10.1016/j.jht.2017.03.008

[19] Salminger S, et al. Functional outcome scores with standard myoelectric prostheses in below-elbow amputees. Am J Phys Med Rehabil. 2019;98(2):125–9. 30153123 10.1097/PHM.0000000000001031

[20] Resnik L, Borgia M, Clark M. Function and quality of life of unilateral major upper limb amputees: effect of prosthesis use and type. Arch Phys Med Rehabil. 2020;101(8):1396–406. 32437692 10.1016/j.apmr.2020.04.003

[21] Eckstein MK, et al. Beyond eye gaze: what else can eyetracking reveal about cognition and cognitive development? Dev Cogn Neurosci. 2017;25:69–91. 27908561 10.1016/j.dcn.2016.11.001 PMC6987826

[22] Just MA, Carpenter PA. A theory of reading: from eye fixations to comprehension. Psychol Rev. 1980;87(4):329. 7413885 10.1037/0033-295X.87.4.329

[23] Wu C-J, Liu C-Y. Refined use of the eye-mind hypothesis for scientific argumentation using multiple representations. Instr Sci. 2022;50(4):551–69. 10.1007/s11251-022-09581-w

[24] Meghanathan RN, van Leeuwen C, Nikolaev AR. Fixation duration surpasses pupil size as a measure of memory load in free viewing. Front Hum Neurosci. 2015;8:1063. 25653606 10.3389/fnhum.2014.01063 PMC4301010

[25] Leigh RJ, Kennard C. Using saccades as a research tool in the clinical neurosciences. Brain. 2004;127(Pt 3):460–77. 14607787 10.1093/brain/awh035

[26] Lindner H, et al. Cognitive load in learning to use a multi-function hand. In: MEC20, Fredericton, New Brunswick, Canada, August 10–13, 2020. (Symposium canceled). University of New Brunswick. 2020.

[27] Franzke AW, et al. Users’ and therapists’ perceptions of myoelectric multi-function upper limb prostheses with conventional and pattern recognition control. PLoS ONE. 2019;14(8): e0220899. 31465469 10.1371/journal.pone.0220899 PMC6715185

[28] Cheng KY, Rehani M, Hebert JS. A scoping review of eye tracking metrics used to assess visuomotor behaviours of upper limb prosthesis users. J Neuroeng Rehabil. 2023;20(1):49. 37095489 10.1186/s12984-023-01180-1 PMC10127019

[29] Bouwsema H, et al. Determining skill level in myoelectric prosthesis use with multiple outcome measures. J Rehabil Res Dev. 2012;49(9):1331–48. 23408215 10.1682/JRRD.2011.09.0179

[30] Parr JVV, et al. Examining the spatiotemporal disruption to gaze when using a myoelectric prosthetic hand. J Mot Behav. 2018;50(4):416–25. 28925815 10.1080/00222895.2017.1363703

[31] Sobuh MMD, et al. Visuomotor behaviours when using a myoelectric prosthesis. J Neuroeng Rehabil. 2014;11(1):72. 24758375 10.1186/1743-0003-11-72 PMC4022381

[32] Kyberd PJ. The influence of control format and hand design in single axis myoelectric hands: assessment of functionality of prosthetic hands using the Southampton Hand Assessment Procedure. Prosthet Orthot Int. 2011;35(3):285–93. 21937574 10.1177/0309364611418554

[33] Onkhar V, Dodou D, de Winter J. Evaluating the Tobii Pro Glasses 2 and 3 in static and dynamic conditions. Behav Res. 2023. 10.3758/s13428-023-02173-7. 10.3758/s13428-023-02173-7 PMC11289326 37550466

[34] Tobii Pro Lab. Tobii Pro AB: Danderyd: Tobii Pro AB; 2022.

[35] Holmqvist K, et al. Eye tracking: a comprehensive guide to methods and measures. Oxford: Oxford University Press; 2011.

[36] Manz S, et al. Using mobile eye tracking to measure cognitive load through gaze behavior during walking in lower limb prosthesis users: a preliminary assessment. Clin Biomech. 2024;115:106250. 10.1016/j.clinbiomech.2024.106250 38657356

[37] Mahanama B, et al. Eye movement and pupil measures: a review. Front Computer Sci. 2022;3:733531. 10.3389/fcomp.2021.733531

[38] Hill W, et al. Upper limb prosthetic outcome measures (ULPOM): a working group and their findings. JPO J Prosthet Orthot. 2009;21(9):P69–82. 10.1097/JPO.0b013e3181ae970b

[39] Gregori V, et al. On the visuomotor behavior of amputees and able-bodied people during grasping. Front Bioeng Biotechnol. 2019;7:316. 31799243 10.3389/fbioe.2019.00316 PMC6874164

[40] Lindner H, Hiyoshi A, Hermansson L. Relation between capacity and performance in paediatric upper limb prosthesis users. Prosthet Orthot Int. 2018;42(1):14–20. 28639478 10.1177/0309364617704802

[41] Parr JVV, et al. Visual attention, EEG alpha power and T7-Fz connectivity are implicated in prosthetic hand control and can be optimized through gaze training. J Neuroeng Rehabil. 2019;16(1):52. 31029174 10.1186/s12984-019-0524-x PMC6487034

[42] Lindner HYN, Linacre JM, Hermansson LM. Assessment of capacity for myoelectric control: evaluation of the construct and the rating scale. J Rehabil Med. 2009. 10.2340/16501977-0361. 19479160 10.2340/16501977-0361

[43] Kuiken T, et al. A Comparison of pattern recognition control and direct control of a multiple degree-of-freedom transradial prosthesis. IEEE J Transl Eng Health Med. 2016. 10.1109/JTEHM.2016.2616123. 28560117 10.1109/JTEHM.2016.2616123 PMC5396910

